# Feeling safer: effectiveness, feasibility, and acceptability of continuous pulse oximetry for people who smoke opioids at overdose prevention services in British Columbia, Canada

**DOI:** 10.1186/s12954-024-00963-6

**Published:** 2024-02-20

**Authors:** Jessica Moe, Tamara Chavez, Charotte Marr, Fred Cameron, Damian Feldman-Kiss, Yueqiao Elle Wang, Jessica C. Xavier, Zahra Mamdani, Roy A. Purssell, Amy Salmon, Jane A. Buxton

**Affiliations:** 1 Department of Emergency Medicine, Diamond Health Care Centre, University of British Columbia, 11 Floor – 2775 Laurel Street, Vancouver, BC V5Z 1M9 Canada; 2 BC Centre for Disease Control, 655 West 12 Avenue, Vancouver, BC V5Z 4R4 Canada; 3 CoVaRR-Net’s Indigenous Engagement, Development, and Research Pillar 7, University of British Columbia, 103-1690 Nelson Street, Vancouver, BC V6G 1M5 Canada; 4 Portland Hotel Society, 9 East Hastings Street, Vancouver, BC V6A 1M9 Canada; 5 SOLID Outreach Society, 1056 North Park Street, Victoria, BC V8T 1C6 Canada; 6 University of British Columbia, 2329 West Mall, Vancouver, BC V6T 1Z4 Canada; 7 BC Children’s and Women’s Hospital, 4500 Oak Street, Vancouver, BC V6H 3N1 Canada; 8 Centre for Health Evaluation and Outcome Sciences, St. Paul’s Hospital, 570-1081 Burrard Street, Vancouver, BC V6Z 1Y6 Canada; 9 School of Population of Public Health, University of British Columbia, 2206 East Mall, Vancouver, BC V6T 1Z8 Canada

**Keywords:** Addiction medicine, Harm reduction, Opioid overdose

## Abstract

**Background:**

Smoking is the most common mode of unregulated opioid consumption overall and implicated in fatal overdoses in British Columbia (BC). In part, perception of decreased risk (e.g., fewer who smoke carry naloxone kits) and limited smoking-specific harm reduction services contribute to overdose deaths. Overdose prevention services (OPS) offer supervised settings for drug use. Continuous pulse oximetry, common in acute care, allows real-time, remote oxygen monitoring. We evaluated the effectiveness of a novel continuous pulse oximetry protocol aimed at allowing physical distancing (as required by COVID-19, secluded spaces, and to avoid staff exposure to vaporized opioids), its feasibility, and acceptability at OPS for people who smoke opioids.

**Methods:**

This was a mixed methods survey study. We developed a continuous pulse oximetry protocol in collaboration with clinical experts and people with lived/living experience of substance use. We implemented our protocol from March to August 2021 at four OPS in BC permitting smoking. We included adults (≥ 18 years) presenting to OPS to smoke opioids. Peer researchers collected demographic, health, and substance use information, and conducted structured observations. OPS clients participating in our study, OPS staff, and peer researchers completed post-monitoring surveys. We analyzed responses using a thematic inductive approach and validated themes with peer researchers.

**Results:**

We included 599 smoking events. OPS clients participating in our study had a mean age of 38.5 years; 73% were male. Most (98%) reported using “down”, heroin, or fentanyl; 48% concurrently used other substances (32% of whom reported stimulants); 76% reported smoking alone in the last 3 days; and 36% reported an overdose while smoking. Respondents reported that the protocol facilitated physical distancing, was easy to use, high satisfaction, improved confidence, improved sense of safety, and that they would use it again.

**Conclusions:**

Continuous pulse oximetry allowed safe physical distancing, was feasible, and acceptable in monitoring people who smoke opioids at OPS.

**Supplementary Information:**

The online version contains supplementary material available at 10.1186/s12954-024-00963-6.

## Background

Smoking is currently the most common mode of unregulated opioid consumption overall and among overdose decedents in British Columbia (BC) [1]. From 2016 to 2021, smoking increased from 29 to 56%, while injection declined from 39 to 20% among people who died of overdose [2]. A 2019 survey of 621 harm reduction clients (22 BC jurisdictions) revealed that 63% preferred smoking substances; 73% of people using heroin reported smoking it [3]. The same survey revealed that 40% of participants exclusively smoked opioids, 19% exclusively injected, 28% both smoked and injected, and 15% used another mode of administration [4]. Reasons that participants choose to smoke opioids compared to other methods were varied: 47.6% identified perceptions of safety (e.g., perceptions of lower risk of overdose, blood borne diseases, infections, feeling that they could better control dosage), 26.6% cited preferences (e.g., preferred the effects of smoking, social aspects), and 25.8% identified not wanting to inject (e.g., do not like to inject, unable to find veins) [5]. Fewer people who smoke opioids carry a naloxone kit [6], reflecting lower perceived overdose risk [7, 8]. However, evidence suggests that overdose risk remains high when smoking opioids [9], exacerbated by fentanyl’s rapid onset regardless of modality [10]. In San Franscisco, fentanyl use by smoking increased and by injection decreased from 2018 to 2020, and overdose mortality increased by 270% during that same timeframe. Although limited conclusions may be drawn from this population-level study, it does demonstrate that smoking fentanyl incurs a high overdose risk [11].

Overdose prevention services (OPS), introduced under Ministerial Order [12] in response to the overdose death-related public health emergency [13], allow supervised drug consumption and prevent fatalities [14]. OPS permitting smoking are limited in BC [15]: in February 2023, only 17 of 45 sites reported inhalation services. Barriers include lack of ventilation [16], concerns about exposing staff to vaporized drugs, and restricting smoking to difficult-to-monitor areas [17]. Despite being designated essential services [18], COVID-19 initially led to decreased OPS use particularly for smoking, contributing to increased overdose deaths [19]. Following COVID-19 restrictions in April 2020, fatal overdoses while people used drugs alone rose to 61%. [1]

OPS rely on non-medical staff (often peers, i.e. people in a community who share lived experience) [20] to identify clients demonstrating opioid toxicity (e.g., drowsiness, decreased respirations), and respond (e.g., stimulation, assisted respirations, oxygen, naloxone) [21]. OPS staff’s ability to identify clients needing intervention is limited by physical distancing, secluded spaces, challenging assessments [22], and increasing contamination of unregulated opioids with benzodiazepines, often exacerbating sedation [23, 24].

Continuous pulse oximetry, a technology routinely used in acute care settings, allows continuous, remote oxygen monitoring, decreasing sedation-related mortality [25, 26], but has not been applied in community settings for people who use drugs. Peers are key to overdose response, and pulse oximeters are a tool that can build their capacity to respond. Preceding the current study, a BC Centre for Disease Control pilot provided portable, non-continuous pulse oximeters for peer workers to apply to clients’ fingers during assessments to non-invasively measure blood oxygen levels. This data aimed to aid peers’ decision-making regarding the need for rescue breaths and naloxone to reverse opioid overdose. Peers reported that non-continuous finger pulse oximeters helped them to identify need for response and to clarify next steps, improving confidence in their decisions. Specifically, non-continuous finger pulse oximeters assisted peers to build competencies in client assessments (e.g., using data to decide on interventions), community health practice (e.g., advocating for clients’ needs), communication (e.g., distributing health information to community members), and professional practice (e.g., functioning as part of a health care team) [27, 28]. Pulse oximetry has also been used in other settings by dedicated healthcare providers monitoring people who use opioids to determine the need for further interventions (e.g., supplemental oxygen, naloxone) [29]. Prior to our study, no OPS in BC were using continuous pulse oximetry. Three of four of our partner OPS were using non-continuous finger pulse oximeters to aid their assessments of and responses to clients showing signs of decreased responsiveness and/or respiratory effort.

Remote monitoring is also a novel concept that gained increasing interest during COVID-19 to empower patients to safely self-monitor in community settings. Such an approach could be applicable for people who use drugs in communities to facilitate safer drug use, however self-monitoring requiring people to apply monitors would be challenging for those experiencing sedation. We introduced a novel continuous pulse oximetry protocol at OPS as a first step to testing the applicability of this technology in a community setting, with hopes that our results could also inform approaches to address remote monitoring challenges. Our protocol is targeted towards people who smoke opioids because we recognized an urgent need to address an increase in fatal overdoses among this specific population. Continuous pulse oximetry could allow physical distancing to improve safety of participating OPS clients and staff by improving monitoring in secluded spaces, decreasing staff exposure to vaporized opioids, and enabling compliance with public health recommendations to prevent COVID-19 transmission. Although unstudied for people who smoke opioids at OPS, continuous pulse oximetry could address an urgent need to adapt OPS services for people who smoke opioids to curb rising overdose deaths.

## Methods

### Aims

We primarily aimed to evaluate the effectiveness of a novel continuous pulse oximetry protocol for people who smoke opioids in allowing physical distancing, its feasibility, and acceptability at OPS in BC, Canada. As a secondary aim, we sought to characterize participants who presented to smoke opioids at OPS, and to elaborate their perceived risks of smoking.

### Study setting

We implemented our protocol from March to August 2021 at four health authority-approved, non-profit, peer-staffed OPS with indoor and outdoor smoking facilities: Overdose Prevention Society in Vancouver’s downtown eastside; Rock Bay Landing (also providing emergency and transitional housing), operated by Victoria Cool Aid Society; Travelodge (a residential site), operated by AIDS Vancouver Island; and SOLID Outreach Society (peer-based health education and harm reduction services) in Victoria.

### OPS clients participating in study

We included adults (≥ 18 years) presenting to OPS to smoke opioids. Participating OPS clients enrolled in our study received a $20 honorarium for participating in the continuous pulse oximetry protocol, and for completing data collection and a post-monitoring survey. Partnering OPS received $10/enrollment to compensate for time and assistance [30]. OPS clients participating in our study could enroll once daily and multiple times on different days.

### Study design

This was a mixed methods survey study. We developed a novel continuous pulse oximetry protocol for use at OPS in collaboration with emergency medicine, public health, and community experts. We employed a participatory/community-based design, collaborating with people with lived/living experience of substance use throughout planning, implementation, analysis, and knowledge translation. We held three planning meetings to co-develop procedures and instruments with the Professionals for Ethical Engagement of Peers (PEEP), a BC Centre for Disease Control provincial peer advisory committee [27, 28]. We engaged OPS staff to identify site-specific needs and facilitate implementation. We recruited and trained peer researchers to implement processes, assist staff to apply continuous pulse oximetry, and recruit OPS clients to participate in our study, obtain consent, and collect data. We obtained peer researcher and OPS input on materials and protocols prior to launch, and regular, ongoing feedback during implementation.

Eligible OPS clients were invited to participate anonymously in our study. Peer researchers reviewed a consent form with OPS clients, who provided verbal and implied consent to proceed by accepting to use the oximetry device and participate in the monitoring protocol. Continuous pulse oximetry was only available to study participants at participating OPS. Our continuous pulse oximetry devices included a sensor taped to study participants’ fingers that transmitted real-time oxygen data to a remote monitor via Bluetooth for OPS staff to view [33]. An alarm sounded if oxygen saturation fell to 90% or below for 15 s, based on evidence that arterial oxygen content steeply declines below this threshold [34].

The analysis was not pre-registered and the results should be considered exploratory.

### Data collection

Peer researchers used standardized Data Collection forms to collect demographics, co-morbidities, substance use history, reasons for smoking, and perceived risks. Peer researchers and OPS clients participating in our study completed Post Monitoring Surveys following each monitoring episode, and OPS staff completed anonymous surveys elaborating their experiences with the monitoring protocol every 2 weeks. Surveys were unique to each respondent group. They assessed experiences, comfort, ease of use, whether the protocol allowed physical distancing, their willingness to use a similar protocol again, satisfaction on a Likert scale of 0–5, and feedback for improvement. We administered surveys at every unique event, or biweekly period for OPS staff, because we wanted to capture event-specific experiences with our monitoring protocol over time (e.g., learnings, challenges, comfort levels). Therefore, surveys could be completed by repeat individuals. Standardized forms with complete variable lists are available in Additional file 1: Appendix S1 and Additional file 2: Appendix S2.

All forms were securely stored at OPS. Trained abstractors transcribed them into a secure, web-based database.

### Data analysis

We summarized respondents’ demographic, health, and drug use characteristics. To account for repeat visits, we identified episodes at the same site on distinct days with complete overlap of age, gender, race, co-morbidities, and participant anonymous identifiers as potential duplicates. We removed these before summarizing individual-level variables, and analyzed event-specific variables among total events.

We organized open-ended survey responses using NVivo [35]. We identified themes using an iterative thematic inductive process. As surveys were anonymous, we did not adjust for repeat surveys from the same individuals. We held seven meetings with two peer researchers to validate themes, and two meetings to discuss interpretation and presentation of findings. We reviewed results and interpretation with partnering OPS and PEEP.

### Ethics

The University of British Columbia Research Ethics Board approved this study (H20-02443).

## Results

We included 599 smoking events (Overdose Prevention Society n = 93; Rock Bay Landing n = 91; Travelodge n = 185; SOLID n = 230). We obtained 599 surveys from participating OPS clients, 511 from peer researchers, and 19 from OPS staff. We included all surveys in our analysis.

### Participating OPS clients’ characteristics

Prior to summarizing characteristics of OPS clients participating in our study, we removed potential duplicates (n = 64). Participating OPS clients’ mean age was 38.5 years (standard deviation 11.3) and 72.9% were male (Table 1). Removed duplicate individuals had similar characteristics to those included.

**Table 1:** Characteristics of OPS clients participating in study (N = 535)

**Age (years; mean [SD])**	38.5 (11.3)
**Gender**	
Cisgender male	390 (73)
Cisgender female	132 (24)
Transgender male, transgender female, non-binary, or two-spirit	4 (1)
Prefer not to answer	9 (2)
**Race/ethnicity**	
White	360 (67)
Indigenous or Metis	119 (22)
Mixed	26 (5)
African-American or Asian	9 (2)
Prefer not to answer	21 (4)
**Housing status**	
House/apartment	34 (6)
Supported housing*	169 (32)
Single room occupancy (SRO) hotel	78 (15)
Shelter	128 (24)
On the street	72 (13)
Other*	33 (6)
Prefer not to answer	21 (4)
**Employment status**	
Full-time	22 (4)
Part-time	72 (14)
***Paid volunteer	77 (14)
Unemployed	309 (58)
Prefer not to answer	55 (10)
**Medical comorbidities**	
None	239 (45)
Mental health (e.g., anxiety, depression, bipolar, post-traumatic stress disorder, attention deficit hyperactivity disorder)	154 (29)
Pulmonary (e.g., chronic obstructive pulmonary disease, asthma)	65 (12)
Cardiac (e.g., arrhythmia, heart murmur)	27 (5)
Infectious disease (e.g., HIV, hepatitis C)	16 (3)
Diabetes	11 (2)
Other***	9 (2)
Prefer not to answer	14 (2)

*Supported housing is subsidized housing for adults, seniors, and people with disability with on-site staff supports

*Other housing included hospital, tent, sailboat, couch-surfing, staying with friends or family, and a combination of accommodations

***“Paid volunteer” positions are not salaried employment but volunteer hours for which people receive remuneration, usually as an honorarium

***Other disorders included chronic pain, neuromuscular, renal, and rheumatologic disorders

### Substance use patterns

At OPS visit, 98% (n = 590) of participating OPS clients reported using “down,” (slang for unregulated opioids including fentanyl and heroin), fentanyl, or heroin, and 48% (n = 290) intentionally mixed substances (32% methamphetamines, cocaine, or other stimulants). Fifty-one percent (n = 308) identified overdose as a smoking-related risk, 76% (n = 456) had smoked opioids alone in the last 3 days, and 215 (36%) reported an overdose while smoking (Table 2). We report additional reported patterns of drug use in Additional file 3: Appendix S3.

**Table 2:** Participating OPS clients’ perceptions of smoking-related risks (N = 599)****

**Smoked opioids alone in the last 3 days**	
Yes	456 (76)
No	119 (20)
Prefer not to answer	24 (4)
Participant-reported perceived risks of smoking opioids****	
Overdose	308 (51)
Death	176 (29)
Addiction	12 (2)
Other health issues	52 (9)
Contaminants (e.g., benzos)	11 (2)
Excessive relaxation	9 (2)
Other drug-related	3 (1)
Arrest/incarceration	65 (11)
Robbery/crime	46 (8)
Losing friends/family/work	40 (7)
**Reasons for smoking opioids alone in the last 3 days**	
Was alone/had no one to use with	89 (20)
Preference	47 (10)
Rules	23 (5)
Not wanting to share	19 (4)
Back-up plan in place*****	5 (1)
Combination	6 (1)
Prefer not to answer	267 (59)
**Have ever overdosed while smoking opioids**	
Yes	215 (36)
No	265 (44)
Prefer not to answer	119 (20)

****Participating OPS clients could choose multiple answers, therefore sum > 599

*****Back-up plans included knowing where to buy safe drugs, having drugs tested, people being in the next room, telling someone before using, knowing one's tolerance, having an emergency application on one's phone, and carrying naloxone

### Effectiveness, feasibility, and acceptability

Participating OPS clients and OPS staff noted that the protocol allowed physical distancing (OPS clients n = 544; OPS staff n = 16), was easy to use (OPS clients n = 483; OPS staff n = 17), improved comfort in monitoring and response (OPS clients n = 503; OPS staff n = 16), and would use it again (OPS clients n = 557; OPS staff n = 16). Most participating OPS clients, OPS staff, and peer researchers were highly satisfied with the protocol (Table 3).

**Table 3:** Effectiveness, feasibility, and acceptability outcomes

**OPS clients participating in study (n = 599)**	
Allowed for physical distancing	544 (92.4)
Easy to use	483 (80.6)
Would use again	557 (93)
Improved comfort in being monitored and responded to	503 (84)
Satisfaction (Least = 1, Most = 5)	
1	3 (0.5)
2	7 (1.3)
3	23 (4.1)
4	142 (25.6)
5	380 (68.5)
**OPS staff (n = 19)**	
Allowed for physical distancing	16 (84.2)
Easy to use	17 (89.5)
Would use again	16 (84.2)
Improved comfort in monitoring and responding to participating OPS clients	16 (84.2)
Satisfaction (Least = 1, Most = 5)	
1	0 (0%)
2	0 (0%)
3	2 (10.5)
4	8 (42.1)
5	9 (47.4)
**Peer researchers (n = 599)**	
Satisfaction (Least = 1, Most = 5)	
1	4 (0.8)
2	7 (1.4)
3	20 (4.1)
4	95 (19.5)
5	362 (74.2)

We identified four themes relating to perspectives and experiences.

#### Ease of use

Participating OPS clients (n = 483) and OPS staff (n = 17) most commonly cited ease of use as the reason for high satisfaction. Multiple participating OPS clients (n = 11) and OPS staff (n = 6) specifically identified that the protocol was *“easy to use”* and*”straightforward.”* One participating OPS client stated that the monitoring protocol was *“simple, very user friendly” (OPS client 193).* Inconspicuous sensors *“[felt] like [they weren’t] there” (OPS client 341),* remote monitors’ *“compact size… [made them] easy to use and carry” (OPS client 378),* and discrete sensors allowed client privacy without sacrificing observation.

#### Efficiency compared to non-continuous oximeters

OPS staff (n = 4) noted that continuous pulse oximetry was more efficient in monitoring than non-continuous oximeters, due to real-time accurate oxygen saturation display and alarms allowing timely overdose recognition and reversal: “*it was faster and more accurate than [a non-continuous] oximeter and at 90% the alarm will sound to signal possible distress*” *(OPS staff 317).*

#### Confidence compared to no oximeter at all

OPS staff (n = 4) reported that accurate real-time monitoring increased confidence by removing uncertainty about participating OPS clients’ respiratory status, and improving assurance that they were making appropriate decisions: *“constant monitoring… [was] beneficial to earlier response and monitoring overdose risk… provides a level of certainty that is not possible with simple observation” (OPS staff 509).*

#### Safety

Many participating OPS clients (n = 64) and OPS staff (n = 4) described that continuous pulse oximetry provided a sense of safety when accessing OPS, stating: *“[it] makes people feel safer” (OPS client 131),* provides reassurance that *"people are monitoring [them]” (OPS client 404),* and *“helps save lives” (OPS client 131).*

Peer researchers reported that *“feedback… was positive. People felt safer when they had the oximeter on and it made them feel seen… [they] feel so much better when they know people are watching and monitoring them; it provides a level of safety when [they] are using” (Peer researcher response during data validation meeting December 8, 2021).*

### Challenges

#### Technical issues with readings, alarms, and monitors

Participating OPS clients (n = 20), OPS staff (n = 2), and peer researchers (n = 40) identified that soil or polish on fingernails sometimes inhibited accurate readings, *“the alarm seems to go off a lot of times for reasons I don't understand” (Peer researcher 40), “the machine… [had] difficulty maintaining connection” (OPS staff 163),* and was *“very sensitive to distance” (Peer researcher 143).*

#### Difficulty applying and using finger sensors

Additionally, participating OPS clients (n = 8) and peer researchers (n = 18) identified difficulty applying the monitors, including that *“to get the oximeter on properly and to work took ages” (Peer researcher 4)* and *“the [tape…] becomes messy*” *(Peer researcher 4)*. Furthermore, finger sensors could be restrictive: *“people can’t move their hands freely. People need to have the ability to move especially if you are doing drugs and preparing drugs” (Peer researcher response during data validation meeting November 10, 2021).*

#### Environmental

Peer researchers (n = 23) and participating OPS clients (n = 33) identified challenges with outdoor inhalation areas, such as poor readings when windy, cold, or clients’ fingers were cool. They expressed a need for privacy via enclosed spaces allowing them to smoke confidentially and with dignity, while also having the reassurance of being monitored.

#### Ongoing education

Ongoing training on how to use monitors and read oxygen levels was important to provide peers the confidence to use them. Peer researchers (n = 5) identified iterative learning: continuous pulse oximetry was *“finicky at first but the more [we use it, we] become more confident in troubleshooting” (Peer researcher response during data validation meeting November 3, 2021)*, and similarly that the monitoring protocol was *“difficult at first, but at three weeks it was a breeze” (Peer researcher response during data validation meeting November 3, 2021).*

We summarize troubleshooting recommendations in Additional file 4: Appendix S4.

### Additional benefits

#### Community engagement

OPS staff (n = 5) and peer researchers (n = 45) reported that the study brought in new community members to participate, creating opportunities to connect with people not previously engaged with OPS. The drivers of increased community engagement were multifactorial, and reflected positive responses towards both our pulse oximetry monitoring and study participation itself: community members expressed interest in being monitored, learning about oximetry, receiving compensation, and meeting with peer researchers. Consistently running the study 2–4 days per week over months helped build community members’ trust by allowing regular interaction with peer researchers. OPS staff reported that the study “*facilitated harm reduction conversations, and helped build rapport” (OPS staff 635),* and *“engagement… has been an incredibly positive aspect of the study… a powerful connection between staff and [clients]” (OPS staff 509).*

#### Meaningful peer involvement

Participating OPS clients (n = 24), OPS staff (n = 9) and peer researchers (n = 44) valued that the project was facilitated by people with lived/living experience. Participating OPS clients highlighted that they felt comfortable with and appreciated being interviewed by peers. Peer researchers felt they could create rapport and establish trust with participating OPS clients, allowing them to provide *“real… [not] fake [answers],”* and to *“speak freely from the heart” (Peer researcher response during data validation meeting November 24, 2021).*

#### Capacity building

Peer researchers (n = 11) reported that their involvement built personal capacity and promoted a sense of purpose, confidence, and self-esteem by allowing them to share knowledge with their community and peers. They benefitted from learning about prevalence of smoking opioids, and incidence of overdose while smoking. OPS staff witnessed that *“peers take a leadership role as [researchers]” (OPS staff 636)* and the study was a *“wonderful chance… to gain more skills and confidence in a new field while also sharing their own knowledge and wisdom” (OPS staff 636).* Participating OPS clients (n = 40) and peer researchers (n = 11) stated that participation was educational, allowing them to learn how to use pulse oximetry and to identify correlations between oxygen saturation and overdoses.

## Discussion

Our study is the first to pilot and observe use of continuous pulse oximetry at OPS, demonstrating that a technology commonly used in hospitals can be successfully applied in a community setting. During COVID-19, continuous pulse oximetry allowed OPS staff to maintain physical distancing while monitoring clients in separate inhalation areas and to intervene at early signs of hypoxemia. Continuous pulse oximetry promoted clients’ sense of security and staff confidence in accurate assessments and response while clients smoked in difficult-to-view areas, also allowing staff to prioritize their safety. We also identified challenges to implementation, including technical difficulties with readings, alarms, and monitors, challenges applying and using finger sensors, environmental conditions impacting reliability, and the need for education. Our study provides important insights into peoples’ smoking perceptions: only 51% perceived overdose as a smoking-related risk and 76% reported smoked opioids alone; conversely, 36% reporting having overdosed while smoking.

Our study demonstrates the importance of flexibly adapting harm reduction measures to address the evolving needs of people who use substances during the unregulated drug poisoning emergency. Our study is a proof-of-principle that medical technology can be adapted to non-traditional settings, and can be effectively used by peers to improve care for people who use substances. Our findings expand on growing evidence supporting remote monitoring to observe patients’ health status in communities [36, 37]. Specifically, continuous pulse oximetry at OPS could improve monitoring of people smoking opioids in secluded areas, decrease staff exposure to vaporized opioids, and decrease transmission of respiratory illnesses (e.g., COVID-19). The challenges we identified indicate a need to carefully monitor implementation and respond specifically to issues that may decrease effectiveness or introduce barriers to use. For instance, from an equity lens, oximetry has been shown to be less reliable among people with darker skin tones [38] and therefore, additional measures to promote safe monitoring may be required for certain ethnic and racial minorities. Our study offers crucial lessons about participatory research strategies: meaningful partnerships with people with lived/living experience of drug use in all aspects of design and implementation [39, 40] and capacity-building [27, 41] were integral to our success. OPS sites guided necessary steps in study implementation to ensure that our oximetry protocol met the needs of participating OPS clients and staff. For instance, responding to participating OPS clients’ request for greater privacy while smoking opioids, SOLID OPS set up a separate space specifically for the project. This new space was facilitated by the remote monitoring capability afforded by continuous pulse oximetry. Our work emphasizes the importance of providing tools to peers at the frontlines of community overdose response, who often experience limited support during a public health crisis.

Pulse oximetry improves monitoring and response to complex overdoses due to an ever-changing unregulated drug supply where clinical observation alone unreliably detects hypoxemia, even when people are watched closely [42]. For instance, contamination of unregulated opioids with benzodiazepines is becoming increasingly common [43]. Opioid and benzodiazepine co-ingestion can additively reduce oxygen saturation, however experience in BC has been that prolonged sedation beyond respiratory depression often occurs [23]. In such cases, staff could avoid risks of unnecessary interventions like bag mask ventilation (aerosolization and infectious disease transmission, including COVID-19) and excess naloxone (precipitating acute opioid withdrawal). Another important implication of our findings is the apparent discrepancy between perceived and actual smoking-related overdose risk, which is reflected in the high prevalence of people smoking alone, corroborating public health data [4, 44]. In a study assessing peoples’ preferences for smoking opioids, 47.6% reported safety-related reasons and 14.0% specifically reported lower perceived overdose risk [5]. In context, our findings support that people adapt their drug use patterns attempting to reduce potential harms. Furthermore, 48% of OPS clients participating in our study intentionally mixed opioids with other substances (32% with stimulants): underlying reasons, including whether concurrent drug use is perceived to decrease overdose risk, requires further investigation. Our findings emphasize need for urgent education to address perceptions of decreased overdose risk due to smoking and to reinforce safer smoking practices (e.g., observed settings, with others, naloxone kits, etc.) [5].

### Future directions

Future research should better elucidate perceived risks and drug use patterns among people who smoke opioids to enable public health messages and interventions to address increasing smoking-related fatal overdoses. Our encouraging findings suggest an urgent need to expand continuous pulse oximetry to additional OPS, broader clientele (e.g., people who use by intravenous routes), multiple clients simultaneously, and poorly monitored settings (e.g., shelters). Given known risks of using substances alone, increasing remote continuous pulse oximetry access in private settings (e.g., apps, wearable devices) [45] should be explored. Future research could also consider increasing access to non-monitored pulse oximetry as an overdose prevention strategy for people who use in the company of others in private or recreational settings.

### Limitations

Our study is limited by implementation in a small subset of OPS. OPS vary in clientele, layouts, and levels of staff training, experience, and comfort. Implementation at other sites would likely face different considerations and challenges. Nonetheless, our inclusion of both peer-based and residential sites improves real-world generalizability and is a strength. Geographic variability in unregulated drug supply composition and regulatory frameworks allowing supervised drug use impact generalizability. Methodologically, because our surveys were anonymous, we were unable to account for repeat responses from the same individuals. Nonetheless, we considered respondents’ experiences with each unique monitoring event (or biweekly period for OPS staff) to be important as they reflected event-specific learning, challenges, and comfort levels with our monitoring protocol, allowing us to analyze experiences over time. We therefore included survey responses from repeat clients as we felt they provided valuable additional information. While our findings suggest that continuous pulse oximetry is promising to facilitate overdose response, its utility depends on other supportive resources (e.g., staff availability and time, scale-up costs), and therefore should be considered in conjunction with upstream public health policy to address root causes of increasing overdose rates.

## Conclusions

Our findings demonstrate that continuous pulse oximetry can improve OPS’ capacity to safely monitor people who smoke opioids, specifically in the context of COVID-19 physical distancing rules and outdoor inhalation sites. In conclusion, continuous pulse oximetry is effective at allowing physical distancing, feasible, and acceptable at OPS, and improves relevance of harm reduction services for people who smoke opioids.

### Supplementary Information

**Additional file 1: Appendix S1.** Data collection form.

**Additional file 2: Appendix S2.** Post-monitoring survey.

**Additional file 3: Appendix S3.** OPS clients' reported patterns of drug use.

**Additional file 4: Appendix S4.** Challenges and troubleshooting recommendations.

**Additional file 5: Appendix S5.** Dedication.

## Data Availability

The datasets used and analyzed during the current study are available from the corresponding author on reasonable request.

## Abbreviations

BC: British Columbia
OPS: Overdose prevention services
PEEP: Professionals for Ethical Engagement of Peers
