# Supplementary material for: Feeling safer: effectiveness, feasibility, and acceptability of continuous pulse oximetry for people who smoke opioids at overdose prevention services in British Columbia, Canada
Source: Harm Reduct J. 2024 Feb 20;21:45. doi: 10.1186/s12954-024-00963-6 (PMC10877878; doi:10.1186/s12954-024-00963-6)
Supplement: Supplementary file 1 — Additional file 1: Appendix S1. Data collection form. [file 12954_2024_963_MOESM1_ESM.docx]

**APPENDIX 1: DATA COLLECTION FORM**

1. We are conducting a research study about monitoring oxygen levels of people who smoke opioids, also known as “down”, at overdose prevention sites. We would like to share information about the research study so you can decide whether you would like to participate. Would that be ok?

- **If no:** Thank you for your time. We are also interested in why one may not be interested in participating in the research, though you do not have to answer. Would you be willing to share your reasons? ________________________________________________________________________________________________________________________________________________________________________________________________________________________________________________________________________________________________________________________________________________________________________________________________________________________________________________________________________________________________________________________________________________________________________________________________

________________________________________________________________________

________________________________________________________________________

Thank you. The overdose prevention site remains available for your use.

- **If yes:** Thank you – our study involves giving you a device that is worn on your wrist during your time at the overdose prevention site, to measure your oxygen levels.

We will now tell you more about the study and if you are interested, we will assist you to complete and sign the consent form

*[RA to explain the study and assist participant in verbal consent]*

*[RA to obtain the following information]*

1. Subject ID: __________________________________________________________________
2. Age: ____________________________________________________________
3. Sex: _____________________________________________________________
4. We’d also like to ask a few questions about you.

**Background Information**

Gender: Do you wish to identify by gender and, if so, how do you identify?

______________________________________________________________________________

Housing status: Where do you currently and usually live?

______________________________________________________________________________Employment status: Are you working and if so what do you do for work? ______________________________________________________________________________

Race/ethnicity: What race or ethnicity do you identify with?

______________________________________________________________________________

Past medical history: Do you have heart, lung or mental health conditions?

______________________________________________________________________________

**Type and amount of opioid use**

What type of opioid or opioids do you knowingly usually use?

___________________________________________________________________________

How much and how frequently do you usually use?

___________________________________________________________________________

Which other drugs do you usually use?

______________________________________________________________________________

When did you last use an opioid? Did you use them alone or with a group of people?

______________________________________________________________________________

What type of opioid or opioids did you think you used at that time?

______________________________________________________________________________

Did you intentionally combined/mixed substances at that time? If yes, which substances?

______________________________________________________________________________How do you usually take your opioid (e.g., by injecting, smoking, or other ways?)

______________________________________________________________________________

🡪 If smoke opioids at any time:

Did you smoke opioids alone within the past 3 days? If so, why?

______________________________________________________________________________

Which risks do you feel you expose yourself to when smoking opioids?

______________________________________________________________________________
