# Supplementary material for: Feeling safer: effectiveness, feasibility, and acceptability of continuous pulse oximetry for people who smoke opioids at overdose prevention services in British Columbia, Canada
Source: Harm Reduct J. 2024 Feb 20;21:45. doi: 10.1186/s12954-024-00963-6 (PMC10877878; doi:10.1186/s12954-024-00963-6)
Supplement: Supplementary file 2 — Additional file 2: Appendix S2. Post-monitoring survey. [file 12954_2024_963_MOESM2_ESM.docx]

**APPENDIX 2: POST-MONITORING SURVEY**

**Study Title:** Preventing Opioid Deaths due to COVID Related Increases in Smoking Illicit Substances

**Principal Investigator:** Dr. Jessica Moe, MD, FRCPC

Department of Emergency Medicine

University of British Columbia

**Contact number:** 604-707-2520 – you can leave a confidential voicemail

Many people who smoke opioids come to overdose prevention sites. The main aim of this study is to see whether a device that measures oxygen levels from a distance will improve how well staff at overdose prevention sites can identify people who are experiencing early signs of overdose. A second aim is to better understand what happens to peoples’ oxygen levels when they smoke opioids.

The purpose of this questionnaire is to gain insights from you about your experiences working in an Overdose Prevention Site with participants using this monitor. By completing this questionnaire, you are consenting to participate in this research.

Participation in this study is optional. All of the information collected from this questionnaire will be kept confidential. Information that contains your identity will remain only with the Principal Investigator and/or designate. All data will be stored on a password-protected, encrypted computer, and original hard-copy data sheets will be stored in a locked cabinet at the BCCDC.

The main risks associated with this study for people who use opioids are related to using opioids or “down,” which may lead to overdose. There may be discomfort in adjusting the oxygen monitoring device but there are no known short or long-term harms from its use.

We hope that the information learned from this study can be used in the future to help other people who use drugs, and to provide support for front-line staff to better monitor and respond to overdoses on site.

If you have any concerns or complaints about your rights as a research participant and/or your experiences while participating in this study, contact the Research Participant Complaint Line in the UBC Office of Research Ethics at 604-822-8598 or if long distance e-mail RSIL@ors.ubc.ca or call toll free 1-877-822-8598. Please reference the study number [H20-02443] when calling  so the Complaint Line staff can better assist you.

**Preventing Opioid Deaths due to COVID Related Increase in Smoking Illicit Substances (Preventing OD CRISIS)**

**Survey of Overdose Prevention Site Staff, Clients, and Research Assistants**

Overdose Prevention Site Staff Name: ___________________________________________

Client Identifier: _____________________________________

Peer Research Assistant Name: ______________________________________

**Question**

We are doing a study to better understand whether measuring the oxygen levels of people who smoke opioids could help overdose prevention site staff to monitor and respond to them in a safe and timely manner.

***Overdose Prevention Site Staff***

1. **What was your experience with the oximetry monitoring?**

**__________________________________________________________________________________________________________________________________________________________________________________________________**

**__________________________________________________________________________________________________________________________________________________________________________________________________**

- 1. **Did you feel comfortable monitoring and responding to clients?**

**__________________________________________________________________________________________________________________________________________________________________________________________________**

**__________________________________________________________________________________________________________________________________________________________________________________________________**

- 1. **How easy were the devices and monitoring protocol to use?**

**__________________________________________________________________________________________________________________________________________________________________________________________________**

**__________________________________________________________________________________________________________________________________________________________________________________________________**

**Did the monitoring protocol allow you to keep physical distanced from clients as recommended?**

**__________________________________________________________________________________________________________________________________________________________________________________________________**

**__________________________________________________________________________________________________________________________________________________________________________________________________**

1. **Based on your experience with the oximetry monitoring today, would you be willing to use it again? Why or why not?**

**__________________________________________________________________________________________________________________________________________________________________________________________________**

**__________________________________________________________________________________________________________________________________________________________________________________________________**

1. **On a scale of 0-5, how satisfied were you with the oximetry monitoring?**

**0****_____1_____2_____3_____4_____5**

**Why?**

**__________________________________________________________________________________________________________________________________________________________________________________________________**

1. **Based on your experience here today, what feedback do you have for us?**

**_________________________________________________________________________________________________________________________________________________________________________________________________**

**__________________________________________________________________________________________________________________________________________________________________________________________________**

**What aspects did you like?**

**_________________________________________________________________________________________________________________________________________________________________________________________________**

**__________________________________________________________________________________________________________________________________________________________________________________________________**

**What aspects did you not like?**

**_________________________________________________________________________________________________________________________________________________________________________________________________**

**__________________________________________________________________________________________________________________________________________________________________________________________________**

**What could we have done better to support you, specifically related to your ability to monitor and respond to clients?**

**__________________________________________________________________________________________________________________________________________________________________________________________________**

**_________________________________________________________________________________________________**

***Client***

1. **What was your experience with the oximetry monitoring?**

__________________________________________________________________________________________________________________________________________________________________________________________________

__________________________________________________________________________________________________________________________________________________________________________________________________

- 1. **Did you feel comfortable being monitored by staff from a distance?**

__________________________________________________________________________________________________________________________________________________________________________________________________

__________________________________________________________________________________________________________________________________________________________________________________________________

- 1. **How easy were the devices to use?**

__________________________________________________________________________________________________________________________________________________________________________________________________

__________________________________________________________________________________________________________________________________________________________________________________________________

1. **Based on your experience with the oximetry monitoring today, would you be willing to use it again? Why or why not?**

__________________________________________________________________________________________________________________________________________________________________________________________________

__________________________________________________________________________________________________________________________________________________________________________________________________

1. **Based on your experience with the oximetry monitoring today, would you recommend it to a friend? Why or why not?**

__________________________________________________________________________________________________________________________________________________________________________________________________

__________________________________________________________________________________________________________________________________________________________________________________________________

1. **On a scale of 0-5, how satisfied were you with the oximetry monitoring?**

**0_____1_____2_____3_____4_____5**

**Why?**

**__________________________________________________________________________________________________________________________________________________________________________________________________**

**_________________________________________________________________________________________________**

**_________________________________________________________________________________________________**

1. **Based on your experience here today, what feedback do you have for us?**

**_________________________________________________________________________________________________________________________________________________________________________________________________**

**__________________________________________________________________________________________________________________________________________________________________________________________________**

**What aspects did you like?**

**_________________________________________________________________________________________________________________________________________________________________________________________________**

**__________________________________________________________________________________________________________________________________________________________________________________________________**

**What aspects did you not like?**

**_________________________________________________________________________________________________________________________________________________________________________________________________**

**__________________________________________________________________________________________________________________________________________________________________________________________________**

**What could we have done better to support you, specifically related to your smoking experience?**

**_________________________________________________________________________________________________________________________________________________________________________________________________**

**__________________________________________________________________________________________________________________________________________________________________________________________________**

**Please ensure Bluetooth chip is returned.**

- **Bluetooth chip returned**
- **Honorarium received**

**Participants initials _____________________________________________________________________**

**Research Assistants initials ___________________________________________________________**

***Peer Research Assistant***

1. **On a scale of 0-5, how satisfied were you with the oximetry monitoring?**

**0_____1_____2_____3_____4_____5**

**Why?**

**__________________________________________________________________________________________________________________________________________________________________________________________________**

1. **Based on your experience here today, what feedback do you have for us?**

**__________________________________________________________________________________________________________________________________________________________________________________________________**

**__________________________________________________________________________________________________________________________________________________________________________________________________**

**What aspects did you like?**

**_________________________________________________________________________________________________________________________________________________________________________________________________**

**__________________________________________________________________________________________________________________________________________________________________________________________________**

**What aspects did you not like?**

**_________________________________________________________________________________________________________________________________________________________________________________________________**

**__________________________________________________________________________________________________________________________________________________________________________________________________**

**What could we have done better to support you, specifically related to your ability to conduct study processes?**

**_________________________________________________________________________________________________________________________________________________________________________________________________**

**__________________________________________________________________________________________________________________________________________________________________________________________________**
