# Supplementary material for: Feeling safer: effectiveness, feasibility, and acceptability of continuous pulse oximetry for people who smoke opioids at overdose prevention services in British Columbia, Canada
Source: Harm Reduct J. 2024 Feb 20;21:45. doi: 10.1186/s12954-024-00963-6 (PMC10877878; doi:10.1186/s12954-024-00963-6)
Supplement: Supplementary file 3 — Additional file 3: Appendix S3. OPS clients' reported patterns of drug use. [file 12954_2024_963_MOESM3_ESM.docx]

**APPENDIX 3**

**SUPPLEMENTARY TABLE: OPS CLIENTS’ REPORTED PATTERNS OF DRUG USE (N=599)**

| Opioids that OPS clients reported using at time of OPS visit**** |  |
| --- | --- |
| Heroin, "Down" or Fentanyl | 590 (98) |
| Hydromorphone | 134 (22) |
| Methadone | 112 (19) |
| Morphine | 66 (11) |
| Oxycodone | 36 (6) |
| Oxycontin | 34 (6) |
| MS Contin | 21 (4) |
| Buprenorphine | 18 (3) |
| Intentionally used opioids with other substances at last use | 290 (48) |
| Methamphetamine | 141 (24) |
| Cannabis | 51 (8) |
| Crack/powder cocaine | 22 (4) |
| Stimulant and cannabis | 24 (4) |
| Another opioid | 16 (3) |
| Other drugs***** | 9 (1) |
| Prefer not to answer | 27 (8) |
| Preferred routes of opioid use**** |  |
| Smoke | 572 (95) |
| Inject | 196 (33) |
| Snort | 43 (7) |
| Swallow | 21 (4) |
| Rectal | 3 (1) |
| Preferred inhalation device when smoking opioids (n=599) |  |
| Stem and/or tin foil | 465 (78) |
| Pipe | 53 (9) |
| Bong | 6 (1) |
| Combination | 26 (4) |
| Prefer not to answer | 49 (8) |

****OPS clients could choose multiple answers, therefore sum > 599

*****Other drugs included multiple stimulants and benzodiazepines.
