# Supplementary material for: Feeling safer: effectiveness, feasibility, and acceptability of continuous pulse oximetry for people who smoke opioids at overdose prevention services in British Columbia, Canada
Source: Harm Reduct J. 2024 Feb 20;21:45. doi: 10.1186/s12954-024-00963-6 (PMC10877878; doi:10.1186/s12954-024-00963-6)
Supplement: Supplementary file 4 — Additional file 4: Appendix S4. Challenges and troubleshooting recommendations. [file 12954_2024_963_MOESM4_ESM.docx]

**APPENDIX 4: CHALLENGES AND TROUBLESHOOTING RECOMMENDATIONS**

**Summary of technical challenges and recommendations**

| **Technical Challenges** | **Recommendations** |
| --- | --- |
| **Remote monitor alarm consistently goes off**  **Difficulties maintaining a Bluetooth connection** | - Wash hands and nails. Dirt, grease, or nail polish on the nail bed can interfere with the readings. - If a wash station is not available, use alcohol swabs to clean hands and nails. - Place the sensor on a nail bed without nail polish, fake nails, or dirt. - Place the sensor so it lays flat and is tightly applied to the fingernail. The red light from the sensor should not be shining through the finger. - Move the individual closer to the remote monitor. - If the sensor did not work on one fingernail, change it to another. - Wait a few (~3) minutes to ensure the oxygen saturation reading comes through. If no reading, turn the remote monitor to the maximum setting. - Put the remote monitor on maximum setting. The setting has to be changed for each individual. |
| **Inconsistent function of the continuous pulse oximetry sensor** | - A blue light appears on the Bluetooth adaptor when working. A red or white light indicates that the sensor is faulty and needs to be replaced. - If the continuous pulse oximetry sensor is faulty, replace the sensor. |
| **Individuals not able to move hands or fingers** | - Helpful to have the individual prepare their drugs before applying the continuous pulse oximetry sensor. - Ensure that the sensor is correctly positioned on the individual’s nail bed. The sensor should lay flat and be tightly applied to the fingernail. |
| **Difficulties placing the sensor on the individual** | - Place the white wrist band around the individual’s wrist first. - Attach the sensor to the longest/widest nail bed (full nail bed) and wrap the adhesive tape around the fingernail for best placement. - Place the sensor so it lays flat and is tightly applied to the fingernail. The red light from the sensor should not be shining through the finger. |

**Summary of environmental challenges and recommendations**

| **Environmental Challenges** | **Recommendations** |
| --- | --- |
| **Weather** | - In the summer months, the outdoor inhalation sites can get too hot as there are no fans or proper ventilation available due to a lack of electrical outlets. Indoor sites with fans and proper ventilation would be ideal. - Too much wind can make it difficult for people to smoke opioids. At colder temperatures, pulse oximeters can be inaccurate because of decreased blood flow to cold hands. Canopy tent sidewalls or a wind blocker can assist with privacy and help to protect from wind while people are smoking outside. |
| **Lack of Privacy** | - There is a need for inhalation sites to include enclosed spaces that allow clients to safely smoke opioids. |
