# Supplementary material for: Feeling safer: effectiveness, feasibility, and acceptability of continuous pulse oximetry for people who smoke opioids at overdose prevention services in British Columbia, Canada
Source: Harm Reduct J. 2024 Feb 20;21:45. doi: 10.1186/s12954-024-00963-6 (PMC10877878; doi:10.1186/s12954-024-00963-6)

**DEDICATION**

We dedicate this study to Brent Donovan, who was instrumental as a peer researcher from SOLID Outreach Society and died in 2022.

From our research coordinator: We are grateful we had the opportunity to know and work with Brent. He was a resource of knowledge and took pride in his work. He had an amazing way of connecting with everyone he met. We always looked forward to visiting SOLID and connecting with Brent. He always brought a smile to our faces.

From a colleague at SOLID: Brent has served as president for the past five years at SOLID. He has worked with people in the street community for over 20years, with many years spent at the Mustard Seed as an outreach and support worker, and more recently doing overdose prevention work. Brent is the familiar face pretty much everyone around town recognizes and is known as the go-to guy when you have a problem you need to sort out or just someone to talk to. In his time as president, Brent has been our main representative on provincial meetings and bodies, doing regular trips to Vancouver for conferences and to assist in the development of similar organizations by and for people with direct experience of drug use in other municipalities.


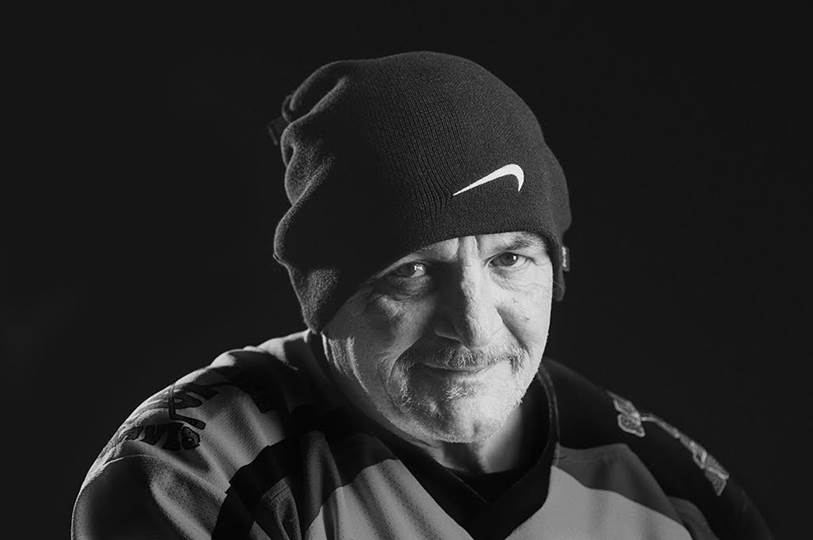

Supplement: Supplementary file 5 — Additional file 5: Appendix S5. Dedication. [file 12954_2024_963_MOESM5_ESM.docx]
